# Urinary Vitamin D Binding Protein: A Potential Novel Marker of Renal Interstitial Inflammation and Fibrosis

**DOI:** 10.1371/journal.pone.0055887

**Published:** 2013-02-11

**Authors:** Katarina Mirković, Carolina R. C. Doorenbos, Wendy A. Dam, Hiddo J. Lambers Heerspink, Maartje C. J. Slagman, Ferdau L. Nauta, Andrea B. Kramer, Ronald T. Gansevoort, Jacob van den Born, Gerjan Navis, Martin H. de Borst

**Affiliations:** 1 Department of Internal Medicine, Division of Nephrology, University of Groningen, University Medical Center Groningen, Groningen, The Netherlands; 2 Department of Clinical Pharmacology, University of Groningen, University Medical Center Groningen, Groningen, The Netherlands; Rouen University Hospital, France

## Abstract

Non-invasive tubulointerstitial damage markers may allow better titration and monitoring of renoprotective therapy. We investigated the value of urinary vitamin D binding protein excretion (uVDBP) as a tubulointerstitial inflammation and fibrosis marker in adriamycin rats, and tested whether uVDBP parallels renal damage and responds to therapy intensification in humans. In adriamycin (ADR) rats, uVDBP was strongly elevated vs controls (CON) already 6 wks after nephrosis induction (ADR: 727±674 [mean±SD] vs CON: 9±12 µg/d, p<0.01), i.e. before onset of pre-fibrotic and inflammatory tubulointerstitial damage, and at all following 6-wk time points until end of follow up at 30 wks (ADR: 1403±1026 vs CON: 206±132 µg/d, p<0.01). In multivariate regression analysis, uVDBP was associated with tubulointerstitial macrophage accumulation (standardized beta = 0.47, p = 0.01) and collagen III expression (standardized beta = 0.44, p = 0.02) independently of albuminuria. In humans, uVDBP was increased in 100 microalbuminuric subjects (44±93 µg/d) and in 47 CKD patients with overt proteinuria (9.2±13.0 mg/d) compared to 100 normoalbuminuric subjects (12±12 µg/d, p<0.001). In CKD patients, uVDBP responded to intensification of renoprotective therapy (ACEi+liberal sodium: 9.2±13.0 mg/d vs dual RAAS blockade+low sodium: 2747±4013, p<0.001), but remained still >100-fold increased during maximal therapy vs normoalbuminurics (p<0.001), consistent with persisting tubulointerstitial damage. UVDBP was associated with tubular and inflammatory damage markers KIM-1 (standardized beta = 0.52, p<0.001), beta-2-microglobuline (st.beta = 0.45, p<0.001), cystatin C (st.beta = 0.40, p<0.001), MCP-1 (st.beta = 0.31, p<0.001) and NGAL (st.beta = 0.20, p = 0.005), independently of albuminuria. UVDBP may be a novel urinary biomarker of tubulointerstitial damage. Prospectively designed studies are required to validate our findings and confirm its relevance in the clinical setting.

## Introduction

The severity of renal interstitial fibrosis has consistently been identified as the strongest predictor of subsequent progressive renal function loss [1]. Early profibrotic changes, on the other hand, might still be reversible [2]. Reliable, non-invasive assessment of tubulointerstitial damage would therefore be of great value in development and monitoring of renoprotective therapy [3].

Vitamin D binding protein (VDBP), also known as group-specific component (GC), is a glycosylated alpha-globulin of 58 kDa [4]. Its main function is to transport vitamin D metabolites through the circulation. VDBP is filtered by the glomerulus and subsequently reabsorbed by proximal tubular cells through receptor-mediated uptake. This process is crucial for retrieval of vitamin D for activation by 1-alpha hydroxylase, which is abundantly present in proximal tubular cells [5]. Tubular reabsorption of VDBP is mediated by megalin and cubulin receptors [6], [7]. Since receptor-mediated uptake of proteins such as VDBP is energy-consuming, tubular injury would be expected to result in urinary VDBP (uVDBP) loss [8]. Indeed, a previous small study suggested that uVDBP is grossly elevated in CKD patients and can be reversed by antiproteinuric treatment [9]. In diabetic subjects, uVDBP is increased compared to non-diabetics, especially when albuminuria is present [10]. Whether uVDBP is related to the extent of tubulointerstitial damage has not specifically been addressed.

We therefore explored the potential role of uVDBP as a marker of tubulointerstitial damage in two independent settings. First we studied the time course of uVDBP in relation to the development of interstitial fibrosis and inflammation in the rat model of adriamycin-induced nephropathy at several time points after induction of nephrosis. We used this model as its protracted time course in the development of renal structural damage allows good resolution over time. Subsequently, in human subjects we addressed the relation between uVDBP and established tubular damage markers in patients with various stages of renal damage, i.e microalbuminuria and overt proteinuria, respectively, as compared to normoalbuminuric controls. We also studied whether intensification of anti-proteinuric therapy would reduce urinary VDBP excretion.

## Materials and Methods

### Ethics Statement

All animal studies as well as clinical studies involving human materials have been conducted according to national and international guidelines. Animal protocols were approved by the Animal Experiments Committee of the University of Groningen, the Netherlands. The clinical studies from which materials were obtained for the current study were approved by the Medical Ethics Committee (Medisch Ethische Toetsingscommissie) of the University of Groningen, The Netherlands, and conducted in accordance with the guidelines of the Declaration of Helsinki. All participants gave written informed consent.

### Animal Model

Male Wistar rats (*n = 90*) were housed in a temperature and light controlled room, with free access to food and water. All rats received normal standard rat chaw containing 2000 UI/kg of vitamin D3.Twenty four hour urine was collected every two weeks by housing in metabolic cages. Surgical procedures took place under isoflurane anesthesia. At the end of the study, the abdominal aorta was cannulated and kidneys were perfused in situ with saline during removal. Proteinuria was measured on a BNII third-generation nephelometer (Dade Behring, Mannheim, Germany).

Rats were assigned to two groups: unilateral adriamycin proteinuric (UAP) nephropathy (*n = 60*), or controls (*n = 30*) [11]. Unilateral adriamycin nephrosis was induced by temporarily clipping the left renal artery through a midline abdominal incision, followed by adriamycin (1.5 mg/kg) injection via the tail vein, as described previously [11]. After 12 min, when adriamycin has been cleared from the circulation [12], the clamp was removed. Control rats underwent the same procedure but were injected with saline. To study renal damage over time, groups of 12 UAP and six control rats were sacrificed at weeks 6, 12, 18, 24, and 30 after surgery. Kidneys were fixed in formalin and embedded into paraffin.

## Patients

For clinical studies, 100 normoalbuminuric and 100 microalbuminuric subjects were randomly selected from Prevention of REnal and Vascular ENd stage Disease (PREVEND), a prospective cohort study investigating the association of albuminuria with renal and cardiovascular disease progression in the general population [13]. In short, between 1997 and 1998, participants of the PREVEND cohort were recruited from 40,856 inhabitants of the city of Groningen, the Netherlands. To obtain a cohort enriched for the presence of elevated albuminuria levels, selection was based on the albumin concentration in a spot morning urine sample. The baseline screening round was completed in by 8,592 participants. Thereafter, participants were invited to visit an outpatient clinic for a medical examination at approximately 3-year intervals. For the present study, data obtained at baseline were used. Anthropometrical, clinical and biochemical data were collected as reported previously [13]. Glomerular filtration rate was estimated (eGFR) with the modified Modification of Diet in Renal Disease (MDRD) formula, taking into account gender, age, race, and serum creatinine concentration. At each screening round two 24-h urine samples were collected after thorough oral and written instructions on how to collect these samples. Urinary albumin concentration was determined in fresh urine samples by nephelometry with a threshold of 2.3 mg/L and intra- and inter-assay coefficients of variation (CV) of 2.2 and 2.6%, respectively (BNII; Dade Behring Diagnostic, Marburg, Germany). Albuminuria is given as the mean of the two 24-hour urine albumin excretion measurements. Urine samples for biomarker measurements were stored at −20°C; plasma samples were stored at −80°C. VDBP was measured in urine samples collected at baseline from 100 randomly selected normo- and microalbuminuric subjects.

Furthermore, urine samples and clinical data were used from a previously performed randomized controlled trial in patients with established non-diabetic chronic kidney disease (CKD) [14]. Briefly, in this study, 52 patients (IgA nephropathy, *n = 15*; FSGS, *n = 14*; membranous nephropathy, *n = 7*; hypertensive nephropathy, *n = 6*; minimal change disease, *n = 2*; unknown, *n = 8*) were subjected to a protocol including a 6-week study period with standard anti-proteinuric therapy (i.e. the angiotensin converting enzyme (ACE) inhibitor lisinopril 40 mg/day and a regular sodium diet of 200 mmol Na+ per day), and another 6-week study period with intensified anti-proteinuric therapy (lisinopril 40 mg/day combined with the AT1 receptor blocker valsartan 320 mg/day and a low sodium diet, target 50 mmol Na+ per day). At the end of each study period, clinical data were recorded and blood and urine samples were collected for analysis. Data and urine samples from both study periods were available for 47 patients for the current post-hoc analyses [14].

### Measurement of Urinary VDBP and Other Biomarkers

After thawing urine and plasma samples were vortexed and subsequently centrifuged (14.000 rpm). The supernatant was used for measurements.Human VDBP was measured with a commercially available sandwich ELISA (Immundiagnostik, catalog # K2314, Bensheim, Germany), according to the manufacturer’s instructions. Briefly, plasma (diluted 1∶40000) or urine (diluted 1∶2–1∶5 for controls and 1∶10–1∶3000 for the other groups depending on the concentration of VDBP) was incubated in a microtiter plate coated with polyclonal anti-VDBP antibodies for one hour. Subsequently, a polyclonal peroxidase-labeled rabbit-anti-VDBP detection antibody was added and incubated for one hour. After washing, tetramethylbenzidine was added as substrate for 15 minutes. After adding a stop solution, absorbance at 450 nm was measured by a spectrophotometer (BenchMark Plus, Bio-Rad Laboratories, Veenendaal, The Netherlands). Using a standard curve generated with VDBP protein as provided by the manufacturer, final VDBP concentrations were calculated. The detection limit of this ELISA is 1.23 ng/ml; intra-assay CV <5.0% for 16 replicate determinations at concentrations of 24.2 and 42.9 mg/dl and inter-assay CV <12.7% for a concentration of 19.3 mg/dl in 14 different assays on two different lots; recovery ranges from 85–116% and linearity was acceptable (r^2^ = 0.998). Rat VDBP was measured using another commercially available ELISA kit (Alpco, Salem, NH), according to the manufacturer’s instructions. Detection limit as provided by manufacturer: 3.125 ng/ml, range 3.125–100 ng/ml. VDBP excretion was calculated from the VDBP concentration in urine collected over a 24-hour period.

The established proximal tubular damage markers kidney injury molecule-1 (KIM-1), beta-2-microglobulin and cystatin C as well as the inflammation markers monocyte chemoattractant protein-1 (MCP-1) and neutrophil gelatinase associated lipocalin (NGAL) were determined in urine and plasma as described previously [15], [16]. Briefly, we developed direct sandwich-enzyme-linked immunosorbent assays using monoclonal coating antibodies and labeled polyclonal detection antibodies on a Maxisorp plate (Nunc, Denmark). The concentration of the analyte was determined spectrophotometrically by conversion of o-phenylenediamine by a horseradish peroxidase label. KIM-1, beta-2-microglobulin, NGAL and MCP-1 antibodies were obtained from R&D systems (Minneapolis, USA). The intra-assay coefficients of variation (CV) of these ELISA’s were 7.4%, 9.7%, 6.8% and 6.8% respectively. Cystatin C was measured by nephelometry (reagents obtained from Siemens [Marburg, Germany]). Samples were diluted to obtain optimal concentration for measurement. All samples were measured in duplicate.

### Immunohistochemistry

Paraffin sections (4 µm) were deparaffinized and stained with periodic acid-Schiff (PAS) to evaluate focal glomerulosclerosis (FGS). For immunostaining, sections were subjected to heat-induced antigen retrieval by overnight incubation in 0.1M Tris/HCl buffer (pH 9) at 80°C. An automated staining system (DAKO Autostainer, Carpinteria, CA, USA) was used to obtain comparable staining results for all slides. Alpha-smooth muscle actin and monocytes/macrophages were detected using murine monoclonal antibodies (α-SMA: clone 1A4, Sigma, St. Luis, MO, USA, and ED1: Serotec Ltd, Oxford, UK, respectively) for 60 min at room temperature. Binding was detected using sequential incubations with peroxidase-labeled rabbit anti-mouse and peroxidase-labeled goat anti-rabbit antibodies (Dakopatts, Glostrup, Denmark) for 30 min. Collagen III was detected using rabbit polyclonal antibody (Col III, Biogenesis, Ltd, Poole, UK) for 60 minutes at room temperature followed by sequential incubations with peroxidase-labeled goat anti-rabbit and peroxidase-labeled rabbit anti-goat antibodies (Dakopatts, Glostrup, Denmark) for 30 min. Peroxidase activity was developed by using 3,3′- diaminobenzidine tetrachloride containing 0.03% H_2_O_2_ for 10 minutes. Counterstaining was performed using Mayer’s hematoxylin [17].

### Quantification of Renal Damage

Sections were examined in a blinded fashion. The severity of FGS was assessed on PAS sections by semi-quantitative scoring of 50 glomeruli per slide on a scale of 0 to 4. FGS was scored positive if collapse of capillary lumina, mesangial matrix expansion, hyalinosis and adhesion of the glomerular tuft to Bowman’s capsule were simultaneously present. If 25% of glomerulus was affected, a score of 1 was adjudged, 50% was scored as 2, 75% as 3, and 100% as 4. The ultimate score per animal was obtained by multiplying the degree of change by the percentage of glomeruli with the same degree of injury and addition of these scores.

The extent of interstitial α-SMA and collagen III protein expression was measured using computer-assisted morphometry. Fifty cortical interstitial fields (200× magnification) were measured with exclusion of arteries and glomeruli and percentage of the stained area was determined. The ultimate score was calculated by the average of all fields per section. Glomerular and interstitial macrophages were counted manually in respectively 50 glomeruli and 50 interstitial fields per section and the mean number per section/animal was calculated.

### Statistical Analyses

Analyses were performed with PASW Statistics 18.0.3 (SPSS, Armonk, NY). Parametric variables are expressed as mean ± standard deviation (SD), whereas non-parametric variables are given as median (interquartile range). Differences between groups were tested using student’s t-test or Mann-Whitney test where appropriate. Linear regression analysis was performed to address the association between uVDBP and parameters of tubulointerstitial damage, both at the time point of sacrifice. These associations were studied in univariate models as well as in multivariate models adjusting for albuminuria to address whether the relation between uVDBP and parameters of structural renal damage depended on albuminuria. In these analyses, all adriamycin animals were considered together in a regression model adjusted for the timepoint after induction of nephrosis. This allowed us to study the relation between uVDBP and renal damage parameters across the spectrum of renal damage. Distribution of the variables was verified by Q-Q plots and histograms. Non-normally distributed variables were transformed to the natural log before entering the regression model. For all analyses a two-sided p<0.05 was considered statistically significant.

## Results

### Rat Unilateral Adriamycin Model

**Figure 1:**
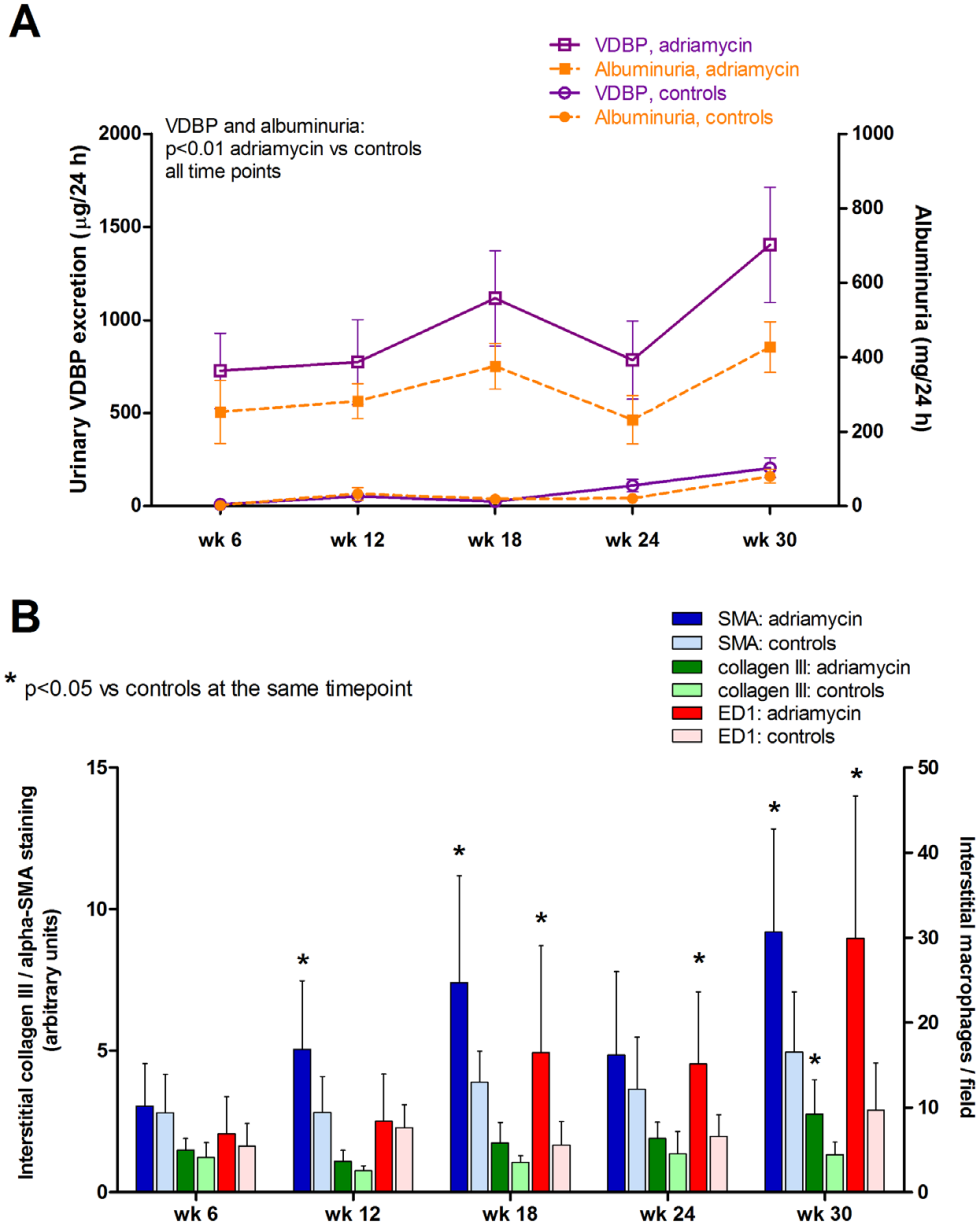
Urinary vitamin D binding protein (uVDBP) and albuminuria levels, and structural renal changes over time in adriamycin and control rats. A. Graphs illustrating the development of albuminuria (orange) and uVDBP (purple) over time in the unilateral adriamycin nephropathy model and in sham-operated control animals. B. Development of tubulointerstitial damage as reflected by interstitial α-smooth muscle actin (blue bars) and collagen III staining (green bars), and interstitial macrophage accumulation (red bars) in adriamycin rats (dark bars) and controls (light bars).

**Figure 2:**
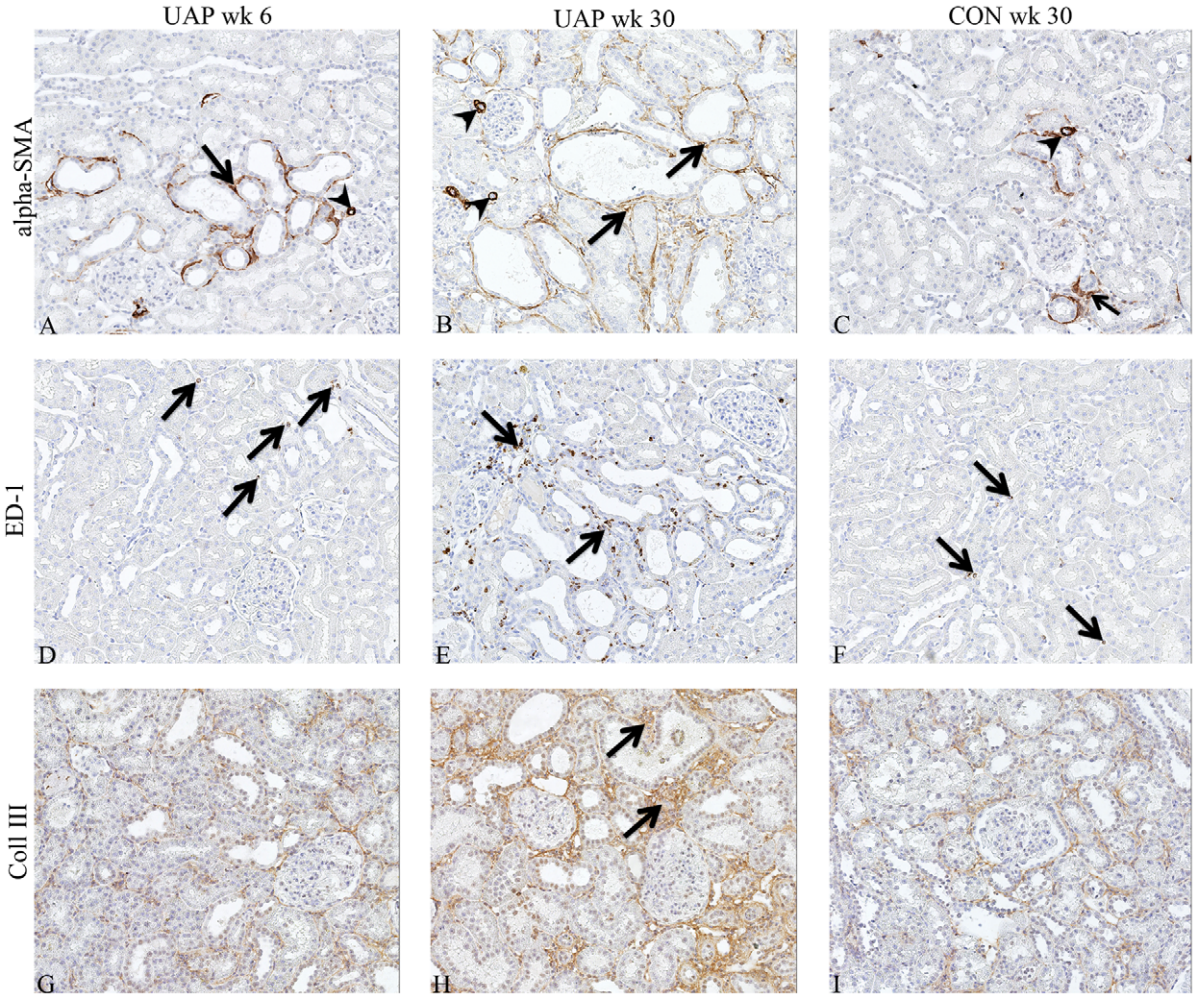
Representative photographs of renal damage markers in adriamycin-exposed and control kidneys. Interstitial α-SMA increased progressively in proteinuric kidneys during follow up (A, B). α-SMA was located around dilated tubules (arrows) and in blood vessels (arrowheads). In control kidneys at week 30, only very limited expression of interstitial α-SMA was present (C). Interstitial macrophage influx increased in ADR animals (D, E). ED-1 positive cells were found mostly around damaged tubules (arrows) and sporadically present in the interstitium of control kidneys (F). Immunostaining of collagen III was found in ADR animals (G, H; arrows), and rarely in controls (I).

The relation between urinary VDBP excretion and structural renal abnormalities in chronic kidney disease was addressed in the rat unilateral adriamycin nephropathy model. This model is characterized by relatively mild albuminuria as only one kidney is affected, followed by the development of renal inflammation, focal glomerulosclerosis (FGS), and tubulointerstitial fibrosis in the affected kidney without concomitant changes in renal function (Table 1), as described in detail previously [18]. Massive urinary VDBP excretion was already present at 6 weeks after adriamycin injection (Figure 1A, (adriamycin: 727±674 µg/d vs controls: 9±12 µg/d, p<0.01), when interstitial alpha-smooth muscle actin (α-SMA) and collagen III expression as well as interstitial macrophage accumulation were still similar to control kidneys (Figure 1B, Figure 2). Also at all other time points until end of follow up at 30 wks (ADR: 1403±1026 vs CON: 206±132 µg/d, p<0.01), VDBP remained elevated in adriamycin rats compared to controls at the same time point after start of the experiment.

**Table 1:** Group characteristics from the experimental study.

		Time				
Parameter	Group	wk 6	wk 12	wk 18	wk 24	wk 30
**Systolic blood pressure (mmHg)**	UAP	129±15	137±11	144±23	151±22	155±15
	CON	121±7	139±12	133±19	151±7	158±20
**Plasma creatinine (µmol/L)**	UAP	5.3±1.3	5.9±1.7	5.7±1.8	6.1±1.4	5.7±0.9
	CON	6.1±0.6	5.8±1	5.3±1	5.6±0.7	5.1±0.8
**Body weight (g)**	UAP	412±17	461±36	496±37	515±36	533±23
	CON	443±28	505±23	505±38	512±20	529±30
**Clipped kidney weight (g)**	UAP	1.7±0.2	1.8±0.2	1.8±0.2	1.8±0.2	1.9±0.2
	CON	1.7±0.2	1.9±0.2	2±0.3	1.8±0.2	2±0.3
**Non-clipped kidney weight (g)**	UAP	1.6±0.2	1.8±0.2	1.9±0.2	1.9±0.3	2.0±0.2
	CON	1.6±0.1	1.9±0.2	2.1±0.1	1.8±0.2	1.9±0.2

ADR – Adriamycin treated rats, CON – control rats, data are presented as mean±SD.

The relation between uVDBP and renal damage markers were subsequently addressed in multivariate regression analysis where all adriamycin rats were analyzed together and data were adjusted for the time point of sacrifice. This approach allowed us to address the relation between uVDBP and renal damage markers across the spectrum of renal damage, analyzing each animal once (at the time of sacrifice). UVDBP was strongly associated with interstitial expression of the pre-fibrotic marker α-SMA and with collagen III expression, as well as with interstitial macrophage accumulation (Table 2). UVDBP was also associated with albuminuria and FGS, but not glomerular macrophage influx. Subsequently the associations between uVBDP and the parameters of renal damage were adjusted for albuminuria in multivariate regression analysis. UVDBP remained significantly associated with α-SMA and collagen III as well as with interstitial macrophage accumulation, independently of albuminuria. The association between uVDBP and FGS, however, did depend on albuminuria (Table 2). Together, these data suggest that uVDBP is already increased before the onset of (early) tubulointerstitial fibrosis and inflammation, and uVDBP increases further in association with increasing tubulo-interstitial damage severity.

**Table 2:** Associations of urinary VDBP with renal histological parameters in the affected kidney in the rat unilateral adriamycin nephropathy model.

	Univariate analysis		Adjusted for albuminuria	
Damage marker	Standardized beta	P	Standardized beta	P
FGS score	0.56	<0.001	0.28	0.52
Glomerular macrophage count	0.15	0.16	0.01	0.97
Interstitial collagen III	0.32	0.005	0.44	0.02
Interstitial macrophage count	0.46	<0.001	0.47	0.01
Interstitial α-SMA protein expression	0.46	<0.001	0.43	0.01

FGS = focal glomerulosclerosis, α-SMA = alpha-smooth muscle actin.

### Clinical Studies

**Figure 3:**
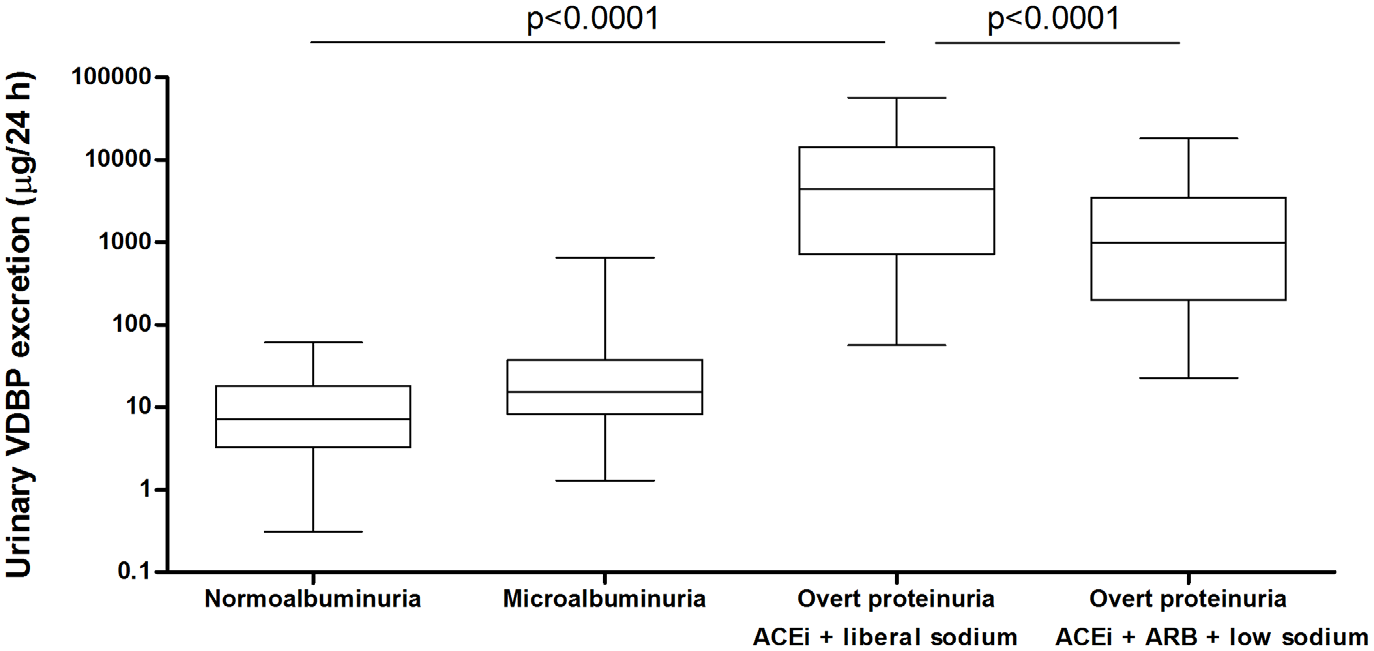
Urinary vitamin D binding protein (uVDBP) levels in patients with normoalbuminuria, microalbuminuria, and overt proteinuria. Box-whisker plots indicating the median, interquartile range, and range of 24-h uVDBP per patient category: general population with normoalbuminuria, general population with microalbuminuria, chronic kidney disease with overt proteinuria treated with ACE inhibitor and liberal sodium diet, and chronic kidney disease with overt proteinuria treated with ACE inhibitor+ARB+low sodium diet. UVDBP increased with albuminuria (first three groups, p<0.001) and responded to intensification of anti-proteinuric treatment (two last groups, p<0.001).

We further characterized urinary VDBP excretion in the clinical setting. Table 3 shows baseline parameters at inclusion for the study groups: subjects with microalbuminuria (*n = 100*) and normoalbuminuric controls *(n = 100)*, both derived from a general population cohort study, and subjects with non-diabetic chronic kidney disease (CKD) with overt proteinuria (*n = 47*). The CKD group was studied during two different treatment periods of 6 weeks, one period with an ACE inhibitor under a liberal sodium diet, and one period with intensified renoprotective therapy: dual renin-angiotensin-aldosterone system (RAAS) blockade (ACE inhibitor and AT1 receptor blocker) under dietary sodium restriction. As shown in Figure 3, uVDBP was low in normoalbuminuric subjects, higher in microalbuminurics, and highest in patients with established CKD (p<0.001). In CKD patients, intensification of anti-proteinuric therapy (dual RAAS blockade and dietary sodium restriction) reduced uVDBP from median 4 mg/day to 1 mg/24 h (p<0.001); however urinary VDBP was still >100-fold increased as compared to normoalbuminuric subjects (median 7 µg/24 h; p<0.001 vs CKD+ACEi+ARB+low sodium), consistent with persistent tubulointerstitial damage despite maximal therapy. When uVDBP was normalized for albuminuria, differences remained similar: patients with normoalbuminuria 0.44 [0.22–0.77] µg/mg; microalbuminuria 0.62 [0.34–1.18] µg/mg; established CKD+ACEi+liberal sodium: 2.44 [1.02–4.51] µg/mg, p<0.001; CKD+ACEi+ARB+low sodium 1.87 [0.92–2.97] µg/mg, p<0.05. Plasma VDBP levels were similar in all groups (normoalbuminuria: 31 [25–42] µg/l; microalbuminuria: 32 [24–43] µg/l; CKD+ACEi+liberal sodium: 39 [38–47] µg/l; CKD+ACEi+ARB+low sodium: 38 [34–42] µg/l; p = NS).

**Table 3:** Baseline characteristics of general population cohort.

	General population cohort		CKD with overt proteinuria		
	Normoalbuminurian = 100	Microalbuminurian = 100	ACEi+liberal sodiumn = 47	ACEi+ARB+low sodiumn = 47	P*
Age (yr)	60±11	60±11	51±13	51±13	−
Male (%)	76	76	83	83	−
Baseline history CVD (%)	22	39	6	6	−
BMI (kg/m^2^)	27±4	29±5	28±4	28±4	−
SBP (mmHg)	140±21	144±21	135±20	122±18	0.001
Blood pressure lowering drugs (%)	25	35	100	100	−
ACEi/ARB (%)	7	8	100	100	−
Cholesterol (mmol/L)	5.2±1.2	5.1±1.2	5.1±1.1	4.9±1.1	0.27
Glucose (mmol/L)	5.1±1.1	5.4±1.2	5.5±0.6	5.3±0.6	0.39
eGFR (ml/min)	78±15	77±15	57±25	48±5	<0.001
Albuminuria (mg/24 h)	14±9	59±51	2191±1734	949±1113	<0.001

* P value calculated by paired t test comparing group on ACE inhibitor (ACEi)+liberal sodium diet versus ACEi+angiotensin receptor blocker (ARB)+low sodium diet. CVD = cardiovascular disease, BMI = body mass index, SBP = systolic blood pressure, eGFR = estimated glomerular filtration rate.

### VDBP is Associated with Established Renal Damage Markers

**Figure 4:**
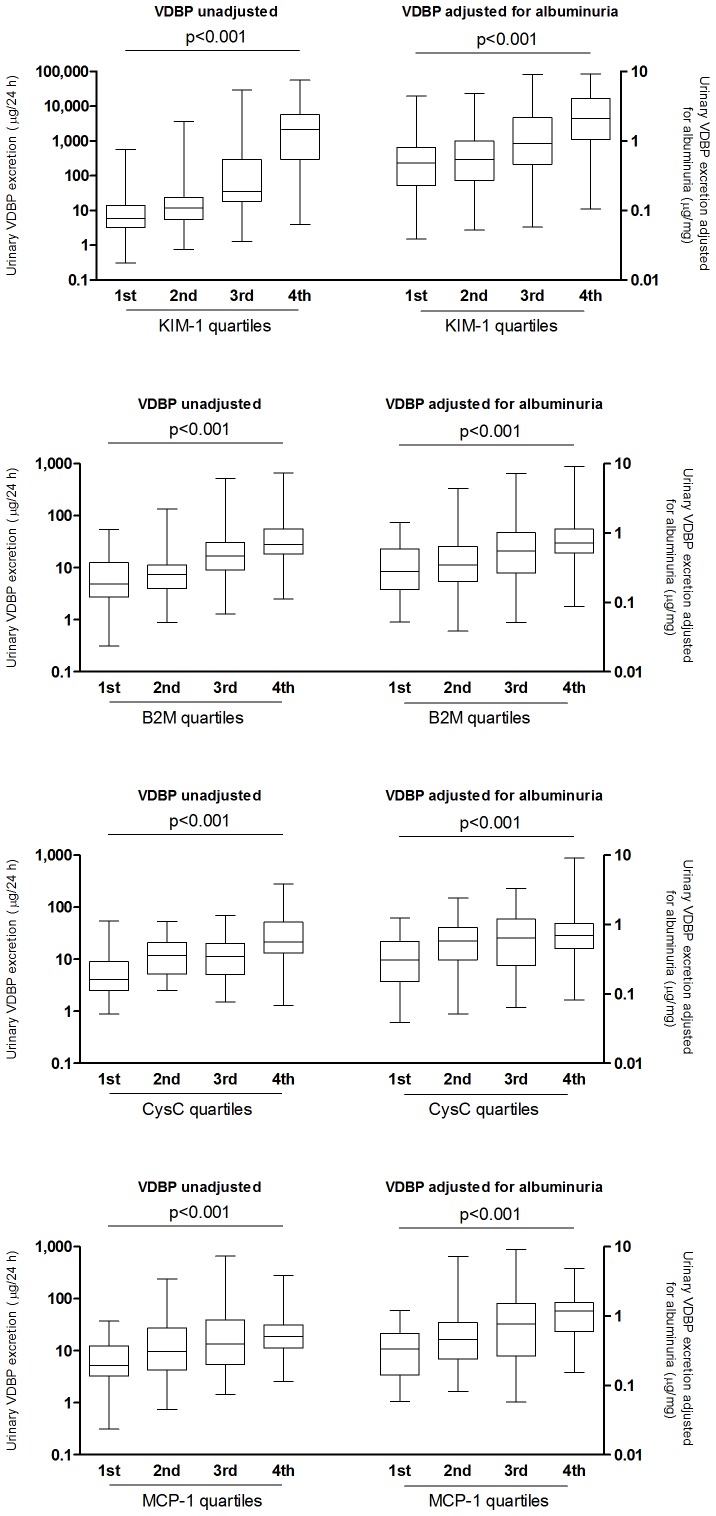
Urinary vitamin D binding protein (uVDBP) and established renal tubular damage markers in patients with renal damage. Box-whisker plots illustrating the relation between uVDBP and established renal tubular damage markers kidney injury molecule-1 (KIM-1) (A), beta-2-microglobulin (B2M) (B), Cystatin C (CysC) (C) and monocyte chemoattractant protein-1 (MCP-1) (D) in normo- and microalbuminuric subjects (both n = 100). The uVDBP is presented per quartile of the established renal damage marker studied. In all panels (A-D), the left four box whisker plots represent the unadjusted VDBP excretion, and the right four box whisker plots represent the uVDBP adjusted for albuminuria. All biomarkers are positively associated with uVDBP, also when VDBP was adjusted for albuminuria (p<0.001, Kruskal Wallis).

The relation between uVDBP and established markers of proximal tubular damage and renal inflammation was further addressed in normo- and microalbuminuric subjects (*n = 200*). UVDBP was strongly associated with urinary KIM-1, beta-2-microglobulin and cystatin C (Table 4 and Figure 4). Of interest, urinary VDBP was also associated with urinary excretion of the inflammation markers MCP-1 and NGAL. These associations were independent of albuminuria (Table 4, Figure 4). Accordingly, the VDBP/albuminuria ratio was associated with KIM-1 (r = 0.50, p<0.001), beta-2-microglobulin (r = 0.26, p<0.001), cystatin C (r = 0.26, p = 0.001), MCP-1 (r = 0.19, p = 0.009) and NGAL (r = 0.16, p = 0.01). Using VDBP/proteinuria ratios yielded similar results. None of the urinary biomarker levels were associated with their plasma levels (data not shown). Neither urinary VDBP excretion nor albuminuria was associated with eGFR.

**Table 4:** Associations of VDBP with renal damage markers across the clinical cohorts.

	Association with uVDBP			
	Univariate		Adjusted for albuminuria	
Damage marker	Standardized beta	P	Standardized beta	p
KIM-1	0.77	<0.001	0.52	<0.001
Beta-2-microglobulin	0.54	<0.001	0.45	<0.001
Cystatin C	0.49	<0.001	0.40	<0.001
MCP-1	0.28	<0.001	0.31	0.001
NGAL	0.20	0.005	0.18	0.00

KIM-1 = kidney injury molecule-1, MCP-1 = monocyte chemoattractant protein-1, NGAL = neutrophil gelatinase associated lipocalin.

## Discussion

To our knowledge this study is the first to demonstrate that urinary VDBP is a biomarker of tubulointerstitial damage, independent of albuminuria, in an animal model of renal damage. In humans, urinary VDBP increased along with the severity of renal damage, was associated with tubular and inflammatory markers independently of albuminuria, and responded to intensification of renoprotective therapy. Yet, the current cornerstone of renoprotective therapy (maximal RAAS blockade combined with dietary sodium restriction) did not achieve normalization of uVDBP, suggesting persistent tubulointerstitial damage in CKD patients. These findings suggest that uVDBP could be developed into a non-invasive urinary marker to monitor tubulointerstitial inflammation and fibrosis.

The urinary loss of VDBP in the setting of renal damage has been reported previously in a rat nephrotoxicity model using urinary proteomics analysis [19], as well as in the setting of chronic kidney disease [9], [10], but its mechanism has not been documented. Interestingly, we found that uVDBP was strongly and consistently elevated in a very early stage of adriamycin-induced nephropathy, even before a detectable increase in the early pre-fibrotic marker α-SMA. UVDBP was also strongly associated with markers of both early and late tubulointerstitial fibrosis (α-SMA and collagen III, respectively) in linear regression analysis adjusted for the time after induction of nephrosis. Of interest, VDBP excretion was also associated with interstitial inflammation, even independent of albuminuria. Its potential as a marker of interstitial inflammation renders VDBP an even more interesting candidate biomarker. The fact that these associations persisted after adjustment for albuminuria suggests that not only protein overload of the megalin/cubulin complex plays a role in urinary VDBP loss. Rather, damaged tubular epithelial cells in areas of tubulointerstitial fibrosis may no longer be able to handle VDBP, resulting in gross VDBP loss into the urine. Indeed, major factors involved in tubulointerstitial fibrosis and inflammation (e.g. TGF-beta and angiotensin II) negatively regulate receptor-mediated endocytosis [20], [21].

We and others reported previously that uVDBP is increased in patients with chronic kidney disease [9], [10], and that it can be modulated by anti-proteinuric treatment in patients [9], although our previous study was of limited sample size. In accordance with our previous study [9], plasma VDBP levels were similar among normo- and microalbuminuric subjects and CKD patients. Our study is in line with previous work showing that uVDBP increases with increasing severity of diabetic nephropathy [10]. Although the combination of optimal RAAS blockade and dietary sodium restriction, an intervention considered optimal for renoprotection, considerably reduced VDBP excretion, it remained >100-fold increased as compared to healthy normoalbuminuric subjects. This suggests that tubulointerstitial damage, considered the final common pathway towards end-stage renal disease (ESRD), persists to a considerable extent despite current best available therapy. This is in line with previous preclinical studies showing progression of pre-fibrotic tubulointerstitial lesions – in spite of optimal reduction of proteinuria – by ACE inhibition and (very strict) dietary sodium restriction [22]. This may explain at least partly why many patients progress to ESRD despite optimal RAAS blockade and dietary sodium restriction – although progression to ESRD is more common in those with high sodium intake [23].

Limitations of our study include the lack of histopathological data in humans to which uVDBP could be related, and the largely cross-sectional design. Although in the animal studies uVDBP was tracked over time, this study does not contain prospective data allowing validation of uVDBP as a biomarker. Urine samples were stored at −20°C which may have influenced VDBP stability. Strengths of our study, on the other hand, include the use of 24 h-urine samples in both human and the animal studies, and the equivocal findings in patients and animals, using two different ELISA systems to detect urinary urinary VDBP. Therefore we believe that our observations can be considered hypothesis-generating and warrant further studies on the predictive value of urinary VDBP in a well-characterized cohort with longitudinal follow-up, as well as studies addressing the associations between uVDBP and histopathological findings in the clinical setting. Furthermore it would be interesting to study uVDBP after kidney transplantation, where interstitial fibrosis also has an important prognostic role.

In conclusion, we demonstrated that urinary VDBP excretion is increased early after renal injury, and is associated with tubulointerstitial inflammation and fibrosis independently of albuminuria in a rat model of proteinuric nephropathy. In humans, uVDBP increased with increasing severity of renal damage, and responded to renoprotective therapy. Yet, persisting uVDBP at >100 times above normal suggested persistent tubulointerstitial damage despite optimal renoprotective therapy. Future studies should address whether urinary VDBP has predictive value for progression of renal function loss, and whether uVDBP is a more suitable early marker of tubulointerstitial damage than the tubular biomarkers currently available, to better guide therapy in chronic kidney disease patients.
